# Interactions among tuberculosis, geographic environment and aerosols: evidence from the Kashgar region of China

**DOI:** 10.3389/fpubh.2025.1519330

**Published:** 2025-03-19

**Authors:** Bo Shang, Chengjing Wei, Chenchen Wang, Yanling Zheng, Liping Zhang

**Affiliations:** ^1^College of Medical Engineering and Technology, Xinjiang Medical University, Urumqi, Xinjiang, China; ^2^Institute of Medical Engineering Interdisciplinary Research, Xinjiang Medical University, Urumqi, Xinjiang, China; ^3^School of Public Health, Xinjiang Medical University, Urumqi, Xinjiang, China; ^4^Xinjiang Uygur Autonomous Region Center for Disease Control and Prevention, Urumqi, Xinjiang, China

**Keywords:** air contaminant, pulmonary tuberculosis, unique topography, spatiotemporal distribution characteristics, source analysis

## Abstract

**Background:**

Aerosols can affect human health through mechanisms like inflammation, oxidative stress, immune dysregulation, and respiratory impairment. In high-pollution areas, airborne particles may promote the transmission of pathogens such as *Mycobacterium tuberculosis*. This study investigates the spatiotemporal distribution of tuberculosis, its association with air pollution, and potential sources in the geographically unique Kashgar region of Xinjiang, encircled by mountains and desert.

**Methods:**

Kriging interpolation and time series observation were used to analyze spatiotemporal trends and identify hot and cold spots of tuberculosis (TB) incidence and air quality in Xinjiang from 2011 to 2023. Kruskal-Wallis and multiple comparisons were applied to assess regional differences. Meteorological clustering and trajectory analysis identified pollutant pathways and potential source areas, with hypotheses proposed for TB transmission routes.

**Results:**

The interaction between tuberculosis, the geographic environment, and aerosols in Xinjiang reveals a consistent spatial distribution of air quality index (AQI) and TB incidence, with overlapping hotspots and cold spots. The incidence rate of tuberculosis is “*n*/100,000.”Southern Xinjiang, shows higher TB incidence (235.31 ± 92.44) and poorer air quality (AQI: 64.19 ± 11.73) compared to Northern Xinjiang (TB: 83.82 ± 21.43, AQI: 53.90 ± 6.48). Significant regional differences in TB incidence (*p* < 0.0001) were confirmed, with post-hoc analyses indicating higher TB rates and worse air quality in Southern Xinjiang. Trajectory and concentration-weighted trajectory (WCWT) analysis identified dust from the Taklimakan Desert as a major contributor to PM_2.5_ and PM_10_ pollution, with values exceeding 150 μg/m^3^ for PM_2.5_ and 400 μg/m^3^ for PM_10_ in key areas like Aksu and Kashgar. The Kunlun and Tianshan mountain ranges serve as barriers that trap migrating dust, while meteorological patterns indicate that dust-laden trajectories extend further into the mountainous areas. This phenomenon exacerbates the spread of tuberculosis (TB) in the high-risk regions of southern Xinjiang.

**Conclusion:**

The study highlights a distinct interaction between TB, the geographic environment, and aerosols in southern Xinjiang. Poor air quality and elevated TB incidence overlap, particularly in Kashgar. Here, dust from the Taklimakan Desert, trapped by the Kunlun and Tianshan mountains, intensifies PM_2.5_ and PM_10_ pollution, further contributing to TB transmission in high-risk areas.

## Introduction

Air pollution, a major global issue, primarily affects the respiratory and circulatory systems of the human body (1–3). Additionally, the impact of air pollutants on the immune system is influenced by respiratory diseases. These effects can impair immune function and increase the risk of various diseases (4–6). Inhaled particulate matter (PM10 and PM2.5) contributes to inflammatory responses, oxidative stress, and immune dysregulation, increasing susceptibility to respiratory diseases like tuberculosis by inducing inflammatory gene expression (e.g., TNF-*α*, IL-1β), suppressing anti-tuberculosis immune responses, and altering the lung microenvironment and immune tolerance (7–13). In 2019, an estimated 10 million new tuberculosis cases and 1.4 million TB-related deaths were reported globally (14). China ranks third in the world in terms of tuberculosis burden, accounting for 8.4% of the global total (15). Therefore, the relationship between the characteristics of air quality pollution and tuberculosis can be studied and analyzed by leveraging the advantage of a large population.

Particulate matter (PM_10_ and PM_2.5_) not only impacts the respiratory and circulatory systems but may also facilitate the spread of infectious diseases like tuberculosis (TB) through complex biological and chemical mechanisms (1, 3). Tuberculosis is an infectious disease caused by *Mycobacterium tuberculosis*. The factors contributing to active tuberculosis are highly complex, encompassing bacterial strains, host immunity, and various social and environmental influences. In heavily polluted areas, such as Xinjiang, China, particulate matter can carry pathogens like *Mycobacterium tuberculosis*, thereby increasing the risk of disease transmission (7). Xinjiang’s unique geographical features, which trap pollutants, further exacerbate the spread of TB in the region (14). Traditional monitoring methods often fail to capture the spatial distribution of pollutants, prompting researchers to employ advanced spatial analysis techniques such as Kriging interpolation and the Potential Source Contribution Function (PSCF) (16, 17). Kriging interpolation estimates pollutant concentrations at unobserved locations based on spatial autocorrelation, offering detailed insights into pollution distribution and source identification (18). PSCF integrates meteorological back-trajectory data with pollutant concentrations to trace the origin of pollutants, particularly in regions with complex topography like Xinjiang, where long-distance pollutant transport is significant (19). These methods, along with Concentration Weighted Trajectory (CWT) analysis, assist researchers in assessing the movement and accumulation of pollutants, and provide critical insights for addressing pollution and tuberculosis transmission in vulnerable areas (20).

**Figure 1:**
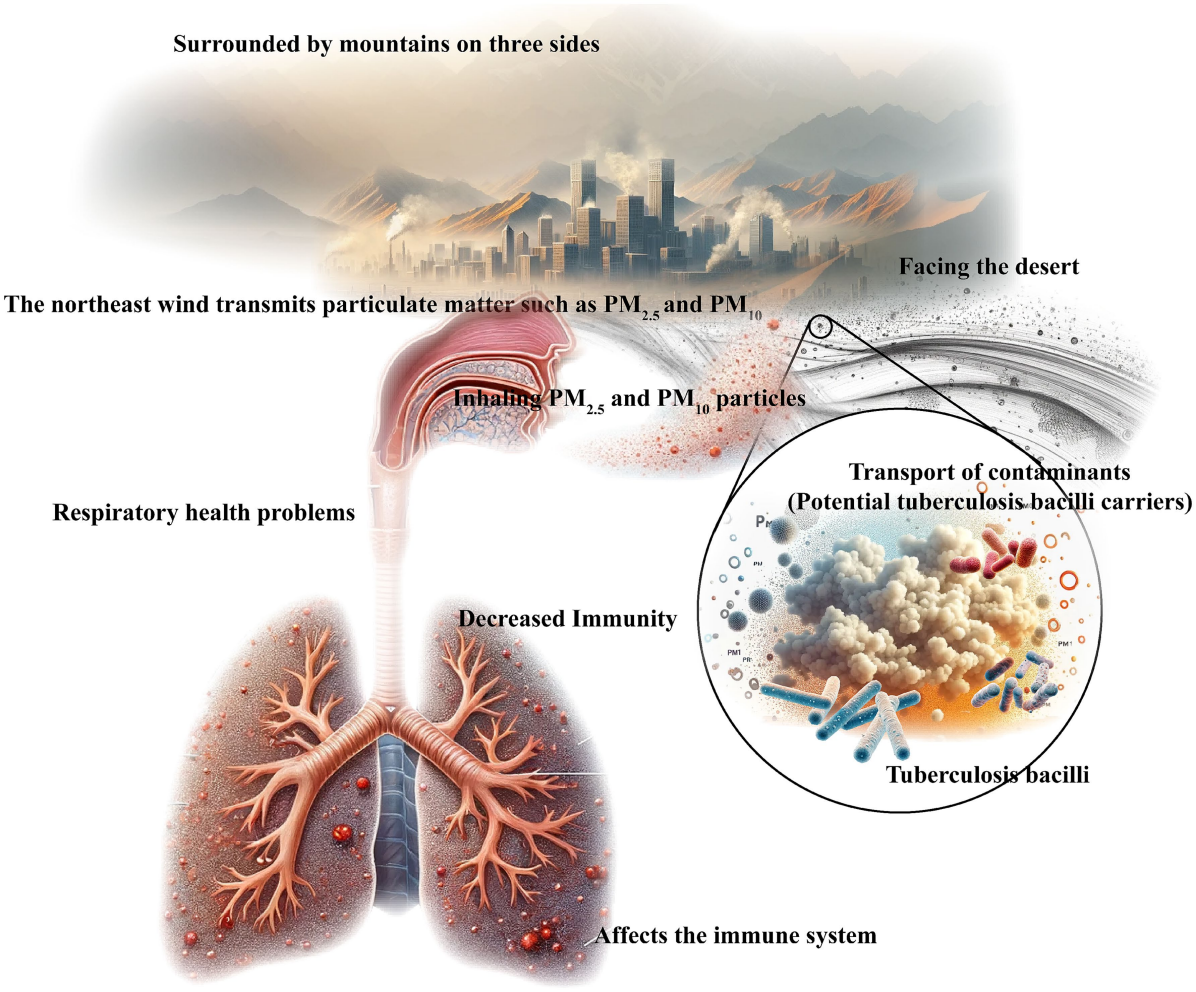
Relationship between geographical environment, transport and distribution of pollutants (potential carriers of tuberculosis bacilli), and human health.

The Xinjiang Uygur Autonomous Region is among the most severely air-polluted areas in China, located in the arid desertification zone of Eurasia. Surrounded by high mountains, the region has limited access to oceanic air flow (21). The Kashgar region, situated in the Xinjiang Uygur Autonomous Region of China, provides a unique geographical setting for examining the relationship between air pollution and tuberculosis bacilli (22, 23). Figure 1 vividly illustrates our research. Surrounded by mountains and adjacent to the vast Taklimakan Desert, Kashgar’s unique topography may contribute to the regional accumulation of air pollutants (24–26).

The Kashgar region in Xinjiang has been recognized as a hotspot for respiratory diseases, including tuberculosis. Tuberculosis is an infectious disease caused by the bacterium *Mycobacterium tuberculosis*, which is primarily transmitted through airborne particles (27). There is concern that airborne particles may act as carriers for *Mycobacterium tuberculosis*, potentially amplifying the transmission of this disease within the population. The Kashgar area, characterized by high population density and relatively crowded living conditions, may thus be at increased risk for the spread of infectious diseases (24, 25). The variations in air pollution levels across different regions of Xinjiang, influenced by numerous factors, correlate with varying disease incidence rates. Single identification will underestimate the impact of air pollutants on diseases. Therefore, this study analyzed potential atmospheric pollution sources and carrier sources of *Mycobacterium tuberculosis* from the source area of pollutants.

Most studies have primarily focused on the isolated associations between air pollutant exposure and DNA methylation or cytokine concentrations, or have employed mediation analysis to explore the intermediary role of DNA methylation or cytokines in the impact of pollutants on tuberculosis risk (26). While systematic reviews and meta-analyses have evaluated the relationship between air pollutants and tuberculosis incidence, no studies have yet examined the interaction between tuberculosis, aerosols, and geographic environment from spatiotemporal dimensions or through long-term, large-scale data tracking (28). This study aims to address this gap. However, the complexity and diversity of environmental factors, combined with the high transmission risk of tuberculosis, present significant challenges in designing experiments to study the intricate interactions between air pollution and TB transmission. In response to these challenges, this study uses a space-oriented approach to explore the relationship between air pollution and TB incidence in Kashgar, a typical region surrounded by mountains on three sides and facing deserts. By simulating the transport of pollutants and analyzing the spatial distribution characteristics of tuberculosis incidence and pollutants, this study aims to elucidate the potential mechanism of air pollution on tuberculosis transmission. Comprehensive data collected over the past decade, including meteorological data, pollutant data, and TB incidence records in Xinjiang, especially during the high TB incidence season (April to June), using advanced analytical techniques such as Kriging interpolation, potential source contribution factor analysis, and concentration weight trajectory analysis. By studying the spatiotemporal dynamics between air quality, tuberculosis incidence, and the unique geographic environment in the Kashgar region, the potential role of geographic factors in pollution patterns and disease transmission dynamics is explored. Understanding the influence of environmental factors on disease epidemiology, predicting key areas that may serve as sources of pollution and contributors to tuberculosis transmission, and revealing how specific geographic regions and meteorological conditions influence disease spread, provide strong support for better control and mitigation of tuberculosis spread and offer valuable insights for future spatiotemporal epidemiological studies.

## Materials and methods

### Disease data

The incidence of tuberculosis in various regions of Xinjiang (One in 100,000) is sourced from the Xinjiang Uygur Autonomous Region Center for Disease Control and Prevention. Rigorous auditing is conducted on all data reports to guarantee data integrity and authenticity. Due to Medical and health conditions, only the incidence data from 2011 to 2022 are available.

### Assessment of ambient air pollutants and meteorological data

Daily statistical data on air pollutants from 2012 to 2023 were obtained from the Environmental Monitoring Station of the Xinjiang Environmental Protection Bureau and the Center for Disease Control and Prevention of the Xinjiang Uygur Autonomous Region. Some data were collected from 153 stations of the China National Environmental Monitoring Center.^1^ The daily monitoring data for SO_2_ (μg/m^3^), NO_2_ (μg/m^3^), PM_2.5_ (μg/m^3^), PM_10_ (μg/m^3^), CO (μg/m^3^), and O_3_ (24 h average concentration (μg/m^3^)) were acquired based on historical monitoring data from air quality monitoring stations across various regions. The air quality index (AQI) is determined by the highest value of the individual air quality index (IAQI) corresponding to each pollutant. The classification of each pollutant is based on the air quality index reference guide (29).

### Meteorological data

The data used in the inverse trajectory model for the second quarter (April to June, the peak incidence period of tuberculosis in the Xinjiang region) from 2015 to 2022 are derived from the Global Data Assimilation System (GDAS) of the National Centers for Environmental Prediction (NCEP). Meteorological information is obtained from the National Meteorological Science Data Sharing Service Platform.^2^ These datasets were initially recorded starting from 2015. This study aligns with the truncation year of tuberculosis statistics and extends until 2022.

### Kriging interpolation method

The Kriging method is a scientific method based on the theory of variogram and structural analysis, which is used for unbiased optimal estimation of regionalized variables in a limited area (30). It is one of the important components of geostatistics and is often used in epidemiological studies to construct and predict disease transmission models. The advantage of this method is that it can take into account the correlation of geographical space, that is, there may be more similar disease transmission patterns between close locations, thus improving the accuracy of prediction. In our study, we used this method to describe the distribution of the incidence of tuberculosis in 2011–2022 and the distribution characteristics of the median size of AQI in 2012–2023. Among them, the ordinary Kriging method is the most used interpolation method in the Kriging method, and its expression is:


(1) $$Z=\overset{m}{\underset{i=1}{\sum }}\lambda_{i}Z_{i},\overset{m}{\underset{i=1}{\sum }}\lambda_{i}=1$$

In Equation 1, Zrepresents the estimated value of the interpolation point, while $Z_{i}$ denotes the measured value of the sample point i. The weight coefficient $\lambda_{i}$ corresponds to the sample point i, and m indicates the number of measured sample points used in the calculation. The weight coefficients for Kriging interpolation are determined based on the semi-variance function, which is expressed as follows:


(2) $$\gamma \left(h\right)=\frac{1}{2N\left(h\right)}\overset{N\left(h\right)}{\underset{r=1}{\sum }}{\left(Z\left(\mu_{i}\right)-Z\left(\mu_{i}+h\right)\right)}^{2}$$

In Equation 2, $N\left(h\right)$ is the number of sampling points with distance h. In the application, it is necessary to select the appropriate semi-variance function model according to the characteristics of the test semi-variance. The selection of weights should ensure that the estimated value Z is unbiased and the estimated variance is smaller than the variance generated by other linear combinations of the observed value. The minimum variance expression of Z is:


(3) $$\sigma_{e}^{2}=\overset{m}{\underset{r=1}{\sum }}\lambda_{i}\gamma \;\left(\mu_{i},\mu_{j}\right)+\phi$$

When the following formula is satisfied, the minimum variance of Z can be obtained.


(4) $$\overset{m}{\underset{i=1}{\sum }}\lambda_{i}\gamma \;\left(\mu_{i},\mu_{j}\right)+\varphi =\gamma \left(\mu_{i},\mu \right)$$

In this Equations 3, 4, m is the number of measured sample points involved in the calculation, ϕ is the Lagrange multiplier, $\gamma \left(\mu_{i},\mu_{j}\right)$ is the semi-variance function between the i sample point and the j sample point, and $\gamma \;\left(\mu_{i},\mu \right)$ is the semi-variance function between the sample point and the trajectory point.

### PSCF and CWT model

Potential Source Contribution Function (PSCF) (31) is a widely used method in recent years for identifying sources of high-concentration pollutants, which helps in understanding the origins, transport pathways, and spatial distribution of atmospheric pollutants (32). In this study, this method was used to indirectly identify the potential source regions of particulate matter and the possible *Mycobacterium tuberculosis* it may carry. This method is based on HYSPLIT regional pollution source identification method. The PSCF at the $\left(i,j\right)$ grid cell is calculated, and its expression is:


(5) $$PSCF_{ij}=\frac{m_{ij}}{n_{ij}}$$

In Equation 5, $m_{ij}$ is the total number of trajectory endpoints in the same grid unit where the measured pollutant concentration exceeds the specified threshold of the pollutant, and $n_{ij}$ is the total number of all trajectory endpoints passing through the $\left(i,j\right)$ grid unit. Regions with high PSCF values indicate a greater contribution to pollutants and may represent high-risk areas for tuberculosis transmission (33). In Equation 6, the PSCF value is multiplied by any weight function $W\left(n_{ij}\right)$ to eliminate the uncertainty in the pixels with smaller $n_{ij}$, so as to better reflect the uncertainty of the median value of these pixels. Based on this, a model was constructed to assign weight to the common spatial distribution of tuberculosis and particulate matter. The weight function is defined as follows:


(6) $$W\left(n_{ij}\right)=\{\begin{array}{l} 1.00\left(n_{ij}>80\right)\\ 0.70\left(25<n_{ij}⩽80\right)\\ 0.42\left(15<n_{ij}⩽25\right)\\ 0.17\left(n_{ij}⩽15\right) \end{array}$$

Weight Potential Source Contribution Function (WPSCF) is often used to analyze the proportion of pollution trajectories in grid cells, but for areas with the same WPSCF value, it is impossible to determine the impact of pollution on the target area accurately. The concentration-weighted trajectory analysis (CWT) method can break through the above limitations and calculate the relative contribution of different source areas, which can better explore the spatial and temporal characteristics of air pollutants (20). By assuming that the particles may carry *Mycobacterium tuberculosis*, the potential source area of epidemic tuberculosis is proposed. The CWT calculation is as follows:


(7) $$C_{ij}=\frac{\sum_{l=1}^{m}C_{l}\tau_{ijl}}{\sum_{l=1}^{m}\tau_{ijl}}$$

In Equation 7, $C_{ij}$ is the weighted average concentration of the $\left(i,j\right)$ grid unit, i is the trajectory index, m is the total number of trajectories, $C_{l}$ is the pollutant concentration corresponding to the trajectory l passing through the network unit $\left(i,j\right)$, and $\tau_{ijl}$ is the time that the trajectory l stays in the grid unit $\left(i,j\right)$. Because the CWT method also has uncertainty, this paper uses the same weight factor $W_{ij}$ as WPSCF to reduce its uncertainty, which called Weight concentration-weighted trajectory analysis (WCWT). The WCWT model was used to better understand how different meteorological conditions and elevation factors, as well as desert environments, affect the distribution of pollutants and the size of potential source areas for TB transmission.

Based on the hour-by-hour data of PM_2.5_ and PM_10_ from 2015 to 2022 in different regions of Xinjiang, the potential source areas of aerosol transmission routes and the potential source areas of tuberculosis transmission were analyzed by WPSCF and WCWT models. The potential source contribution factor and concentration weight trajectory of PM_2.5_ and PM_10_ are calculated by multiplying the potential contribution factor and concentration weight trajectory by the weight factor, respectively. Based on the calculation results of the WPSCF model, we divided the potential source areas into mild (0–0.3), moderate (0.3–0.7), and severe (0.7–1.0) pollution source areas. Combined with the spatial distribution of aerosol and tuberculosis incidence, and based on the WCWT model, we defined areas with particulate matter concentrations below 50 μg/m^3^ as low contribution source areas, 50–150 μg/m^3^ as moderate contribution source areas, and above 150 μg/m^3^ as high contribution source areas. This was done to explore the impact of particulate matter source areas on tuberculosis transmission (34).

### Statistical analysis

Kriging interpolation and time series observations were used to analyze spatiotemporal trends and identify hot and cold spots of tuberculosis (TB) incidence from 2011 to 2023 and air quality in Xinjiang from 2010 to 2022. A predictive model for TB incidence and AQI was constructed using quadratic fitting, and model performance was evaluated using the R^2^ metric and *p-*value. The significance level was set at *α* = 0.05.

Spatial autocorrelation was analyzed for TB incidence and air quality in Xinjiang using Moran’s I index, where positive values near 1 indicate clustering and negative values near −1 suggest dispersion. A z-score of 7.49886, greater than the critical value of 1.65, confirmed significant clustering. Hotspot and cold-spot areas were identified based on 90, 95, and 99% confidence intervals.

Local spatial autocorrelation was assessed using the Gi* statistic, where values near 1 indicate strong clustering and near 0 suggest no significant clustering. Combined analysis of Moran’s I and General G index pinpointed Southern Xinjiang as a hotspot for high-value clustering, revealing spatial heterogeneity in TB incidence and air quality. All analyses were performed using ArcGIS 10.8.2 software (ESRI Inc., United States).

To assess regional differences in TB incidence and air quality, non-parametric statistical tests were conducted. Specifically, the Kruskal-Wallis test was applied to examine the significance of differences between groups. Multiple comparisons were used to further explore and highlight significant regional disparities between different locations within Xinjiang.

Meteorological clustering and trajectory analysis were carried out to identify the pathways of pollutants and potential source areas contributing to air pollution in the region. Using GADS meteorological data and hourly PM_2.5_ and PM_10_ concentration data from 2015 to mid-2022, backward trajectory simulations were conducted to model the movement of atmospheric pollutants over a 72-h period. The simulations were performed at a height of 500 meters and at four daily intervals (00:00, 06:00, 12:00, and 18:00 UTC).

The TrajStat 1.4.9 plug-in in Meteoinfo software was used for clustering and analyzing air mass trajectories across different seasons. The air mass trajectories were categorized into five types based on their movement patterns. Additionally, PM_2.5_ and PM_10_ pollution trajectories were classified according to the secondary standard limits outlined in the “Ambient Air Quality Standard (GB3095-2012) (35)”, which sets thresholds of 75 *μ*g/m^3^ for PM_2.5_ and 150 *μ*g/m^3^ for PM_10_. The number of pollution trajectories for each pollutant was calculated to assess the transport pathways of these pollutants in relation to TB transmission.

Finally, based on the identified pollution pathways and source areas (WPSCF and WCWT), hypotheses were proposed regarding the potential transmission routes of TB, predicting the source areas and the contribution levels of tuberculosis in this region.

## Results

### Overview of the research area

**Figure 2:**
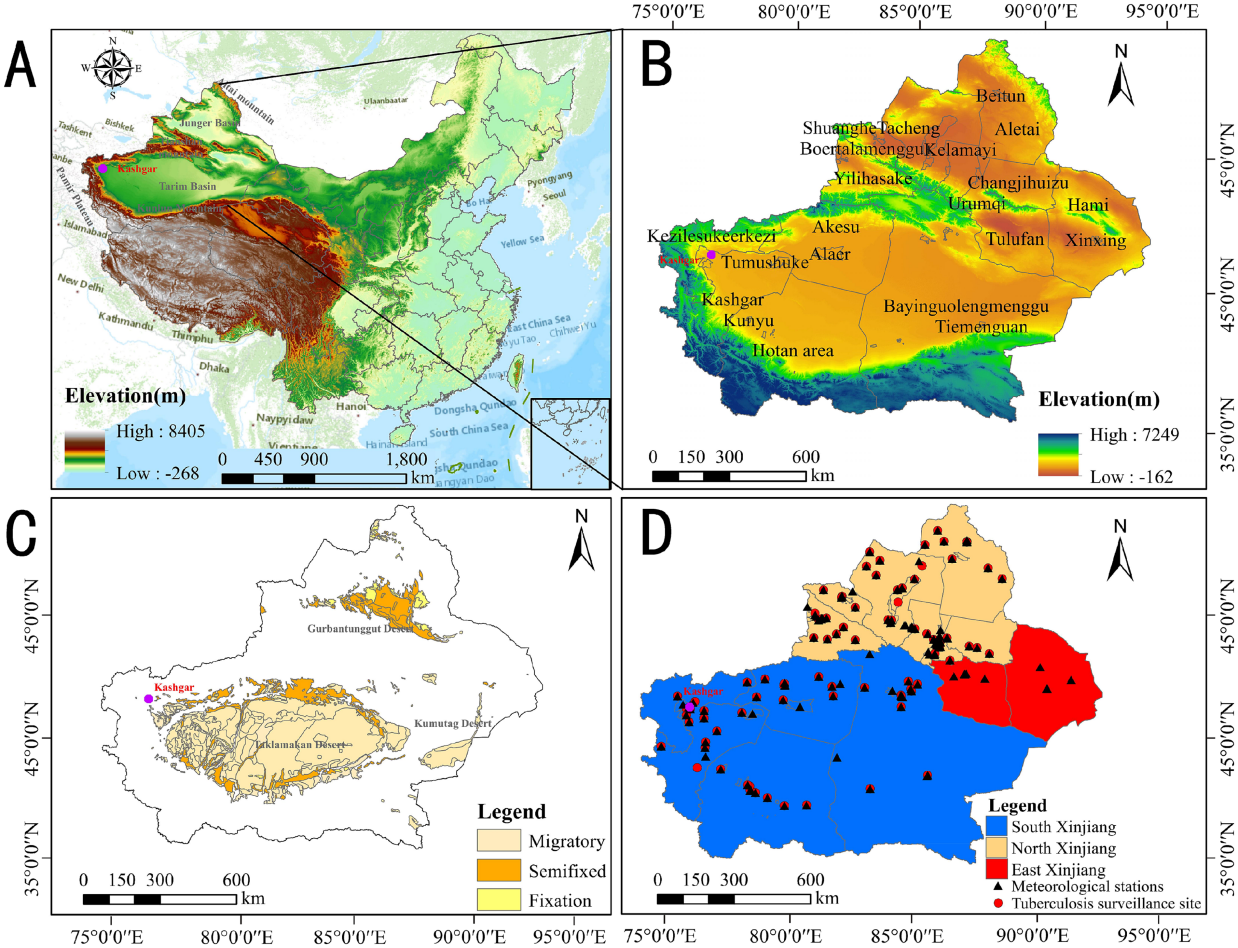
**(A)** Geographical location, **(B)** altitude, **(C)** geographical environment, and **(D)** regional division distribution of site locations in Xinjiang.

Figure 2 shows the geographical, administrative, and environmental distribution, along with site locations in northeast and southwest Xinjiang. Located in northwest China, Xinjiang is highlighted by a purple dot marking Kashgar, the focus of our analysis. Our Kriging interpolation sites include meteorological observation sites (red circles) and tuberculosis monitoring sites (black triangles). The region is divided into southern, northern, and eastern Xinjiang based on local customs.

Kashgar, in southwest Xinjiang, is surrounded by the Tianshan Mountains, Kunlun Mountains, and Pamir Plateau, and bordered by the Taklimakan Desert. Xinjiang has three main desert zones: Taklimakan, Gurbantünggüt, and Kumtag, classified as migratory, semi-fixed, and fixed deserts. Migratory Deserts: The Taklimakan Desert, in the Tarim Basin (Southern Xinjiang), is characterized by shifting sand dunes and harsh, arid conditions. It extends from Hotan to near Aksu and Korla, with highly mobile dunes. Semi-fixed Deserts: The edges of the Taklimakan near Kashgar, Aksu, and Hotan have semi-fixed dunes with some stabilizing vegetation. Parts of the Kumtag Desert, extending east toward Lop Nur (Eastern Xinjiang), are also semi-fixed. Fixed Deserts: The Gurbantünggüt Desert, in Northern Xinjiang’s Junggar Basin, features stabilized sand dunes due to sparse vegetation. The northern Taklimakan and parts of the Kumtag Desert also show characteristics of fixed deserts. Overall, Xinjiang’s deserts vary in dune mobility, with the Taklimakan dominant in the south and the Gurbantünggüt more stable in the north.

The descriptive statistics for both tuberculosis incidence and air quality index (AQI) across four regions of Xinjiang (Eastern, Northern, Southern, and overall Xinjiang) are presented in Table 1. The incidence rate of tuberculosis is “n/100,000.”

**Table 1:** Descriptive statistics for tuberculosis incidence and AQI.

Region	TB incidence (Mean ± SD)	95% CI	AQI (Mean ± SD)	95% CI
Eastern	70.02 ± 21.61	56.97–83.08	52.73 ± 6.30	48.73–56.74
Northern	83.82 ± 21.43	70.87–96.77	53.90 ± 6.48	49.78–58.02
Southern	235.31 ± 92.44	179.45–291.18	64.19 ± 11.73	56.73–71.64
Xinjiang	166.62 ± 58.04	131.54–201.69	58.27 ± 8.03	53.17–63.38

### Time series analysis of pollutants and pulmonary tuberculosis

The analysis of data for the Air Quality Index (AQI) and pulmonary tuberculosis (TB) incidence did not reveal any significant correlation or regression between the two variables. Both correlation analysis (*p* > 0.05) (Supplementary Table S1) and regression analysis (*p* > 0.05) (Supplementary Table S2) indicated a weak association, suggesting that changes in AQI have a limited impact on TB incidence.

**Figure 3:**
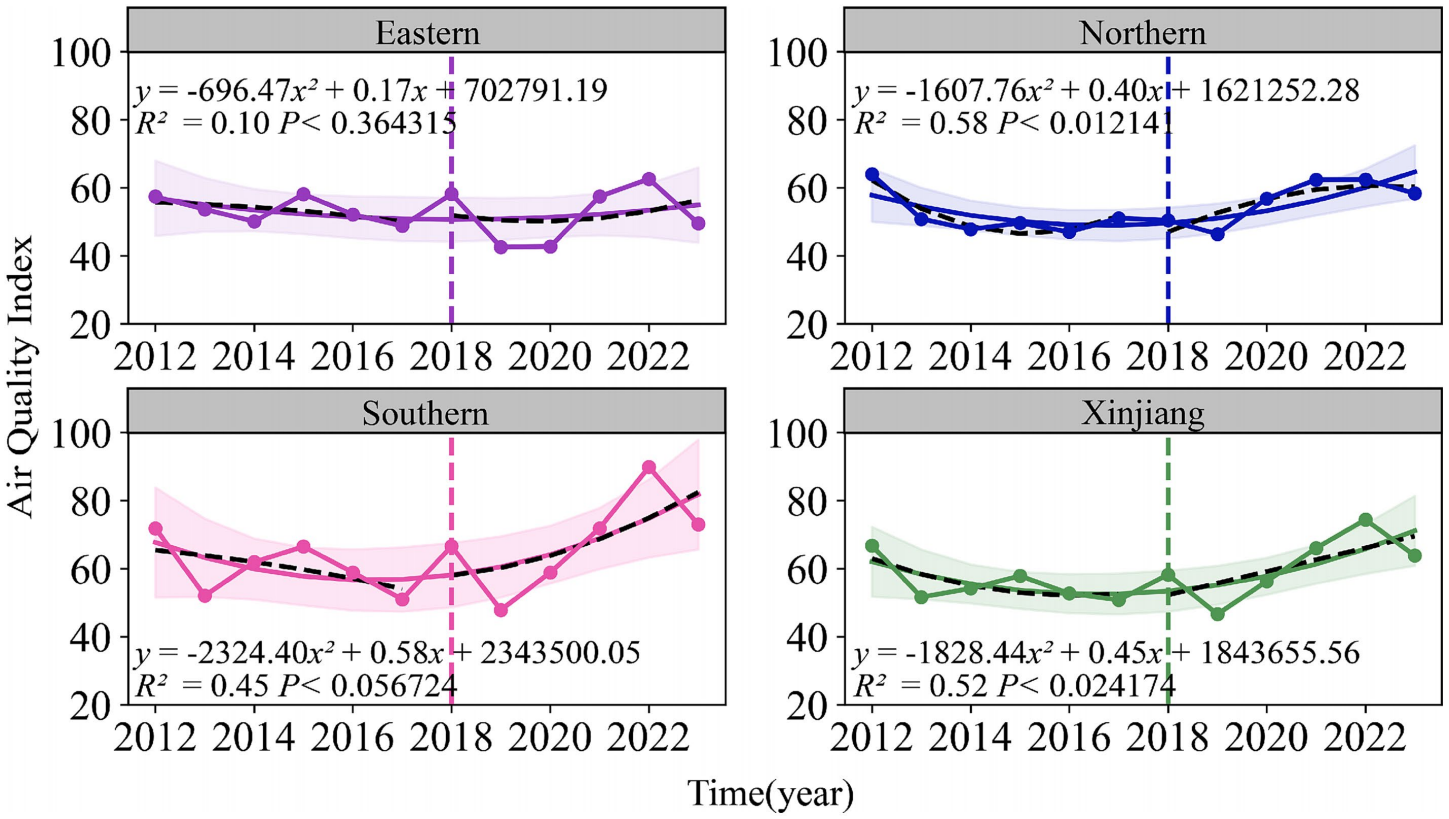
Temporal trends of air quality index (2012–2023) and tuberculosis incidence rates (2010–2022) in eastern, northern, southern Xinjiang, and Xinjiang overall.

**Figure 4:**
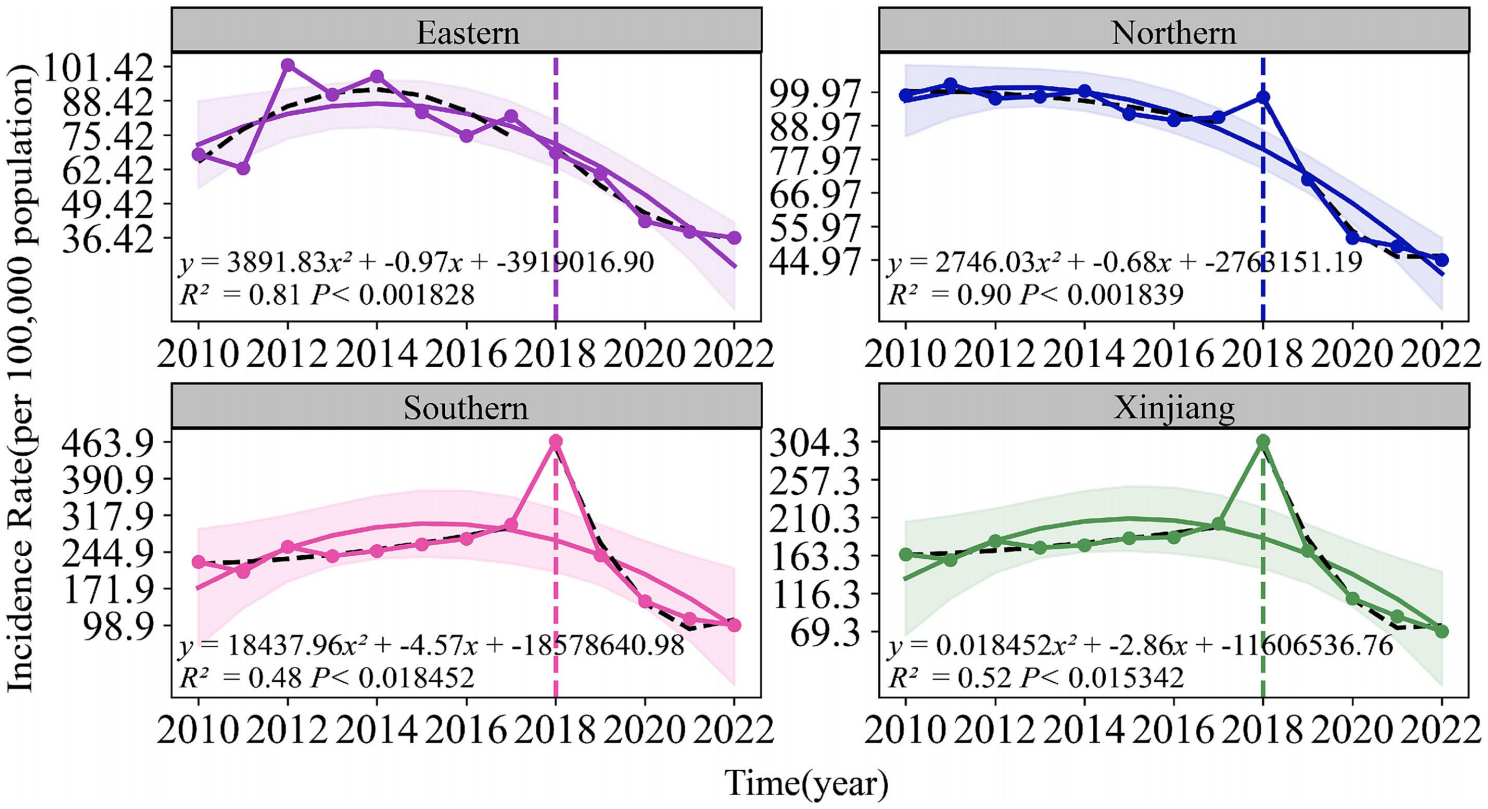
Temporal trends of air quality index (2012–2023) and tuberculosis incidence rates (2010–2022) in eastern, northern, southern Xinjiang, and Xinjiang overall.

As shown in Figures 3, 4, both the Air Quality Index (AQI) and tuberculosis (TB) incidence in Xinjiang exhibited significant trends from 2012 to 2023. Both AQI and TB incidence declined from 2012 to 2018, followed by an upward trend in AQI and a sharp decline in TB incidence after 2018, mainly due to active interventions by the government and health authorities. Following the TB outbreak in 2018, disease control departments learned from the incident and implemented proactive measures in collaboration with the government and healthcare sectors. The relationship between the pandemic’s lockdown measures and the decline in tuberculosis cases lies in the strict control of movement and reduced public interaction, which limited the spread of infectious diseases, including TB. The lockdown and isolation measures likely contributed to a decrease in TB transmission, as they reduced the opportunities for individuals to be exposed to infected people, especially in crowded settings.

There were significant regional fluctuations in air quality, particularly in Southern Xinjiang, which saw high AQI levels in 2012, 2015, 2018, and 2022, indicating severe air quality deterioration. TB incidence also showed notable regional differences, with Southern Xinjiang experiencing a sharp increase from 250 cases per 100,000 in 2015 to over 450 cases per 100,000 in 2018, followed by a steady decline, returning to lower levels by 2022. Meanwhile, Eastern and Northern Xinjiang consistently maintained the lowest TB incidence rates during the study period.

The fitted curve in the figure indicates that the AQI has shown a long-term upward trend, which is associated with the industrialization and urbanization of the region. Among the models, the predictions for Southern and Northern Xinjiang are relatively accurate, with *R*^2^ values of 0.45 and 0.58, respectively. The prediction model for the incidence rate of tuberculosis shows even higher accuracy, particularly in Northern Xinjiang, where R^2^ reaches 0.90, followed by Eastern Xinjiang with an R^2^ of 0.81. The fitting results for all regions, except Eastern Xinjiang, are statistically significant (*p* < 0.05).

**Figure 5:**
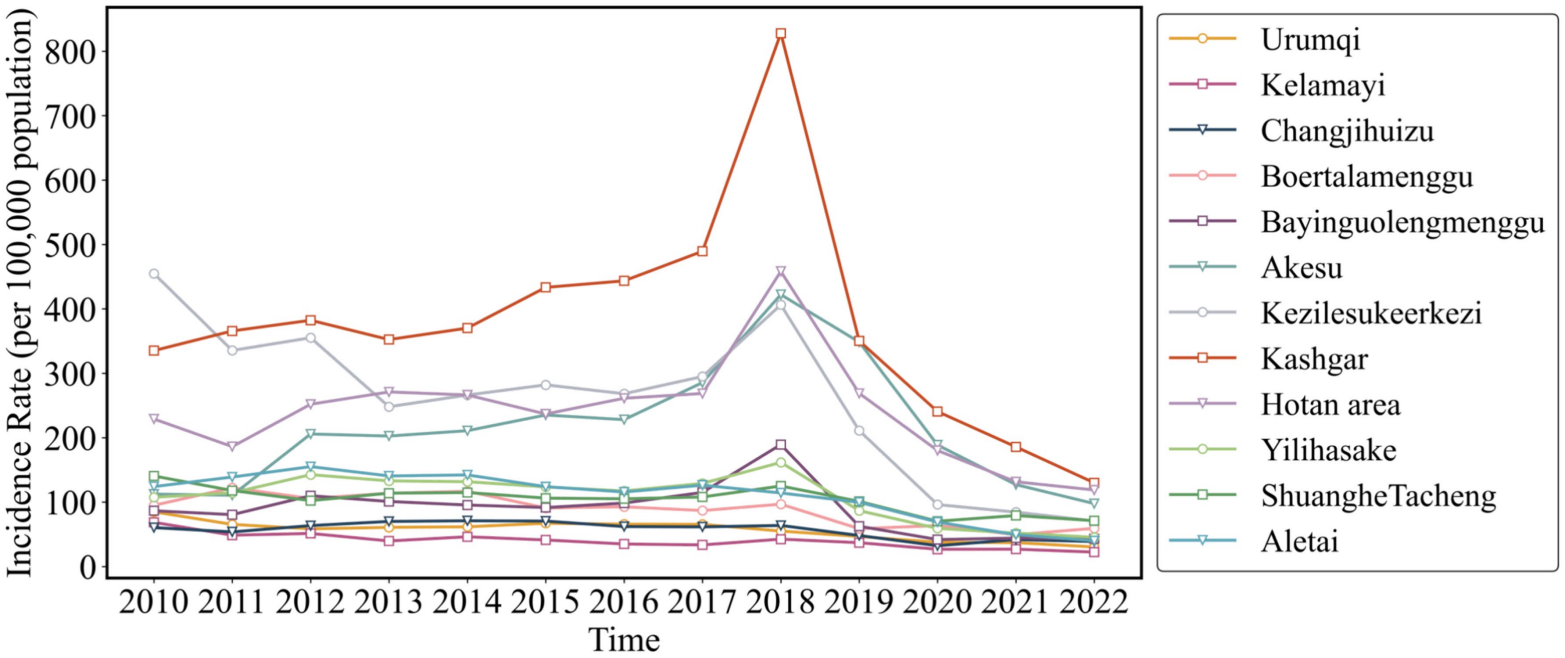
Incidence trend of pulmonary tuberculosis in Xinjiang Uygur Autonomous Region from 2010 to 2022.

Figure 5 illustrates that from 2010 to 2022, areas with high incidence of pulmonary tuberculosis in Xinjiang include Kashgar, Hotan, Akesu, and Kezilesu (Southern Xinjiang), while Kelamayi (Northern Xinjiang) has a low incidence. The correlation between AQI distribution (2013–2023, Figure 6) and tuberculosis incidence is evident in Southern Xinjiang, where higher pollution aligns with higher tuberculosis rates. In contrast, the relationship between AQI and low-incidence areas, like Northern Xinjiang, is less clear. In 2018, the tuberculosis incidence from high to low was Kashgar, Hotan, Akesu, and Kezilesu, mirroring the AQI pattern. From 2010 to 2022, the average tuberculosis incidence in Xinjiang was 166.66 per 100,000 people, with Kashgar reaching 377.43. The top counties-Yingjisha, Zepu, and Maigaiti (Southern Xinjiang)-had the highest rates. Severe air pollution in Kashgar, particularly from 2013 to 2023, correlates with the high incidence of tuberculosis cases. Referring to the tuberculosis trend in Kashgar from 2010 to 2022 (Figure 7), Tashkurgan (Southern Xinjiang) had the lowest prevalence, likely due to its sparse population in a desert area, while other parts of Kashgar remain highly affected.

**Figure 6:**
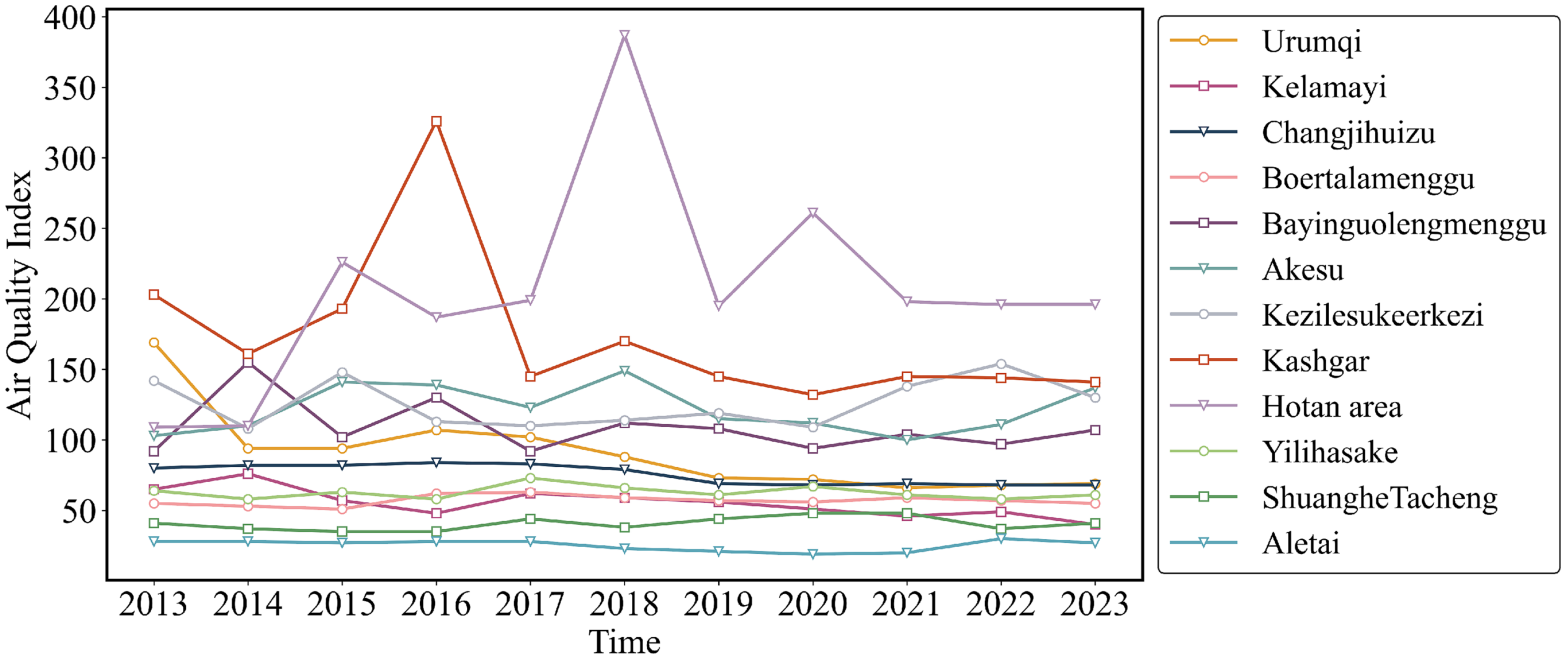
Time distribution characteristics of AQI in Xinjiang Uygur Autonomous Region from 2013 to 2023.

**Figure 7:**
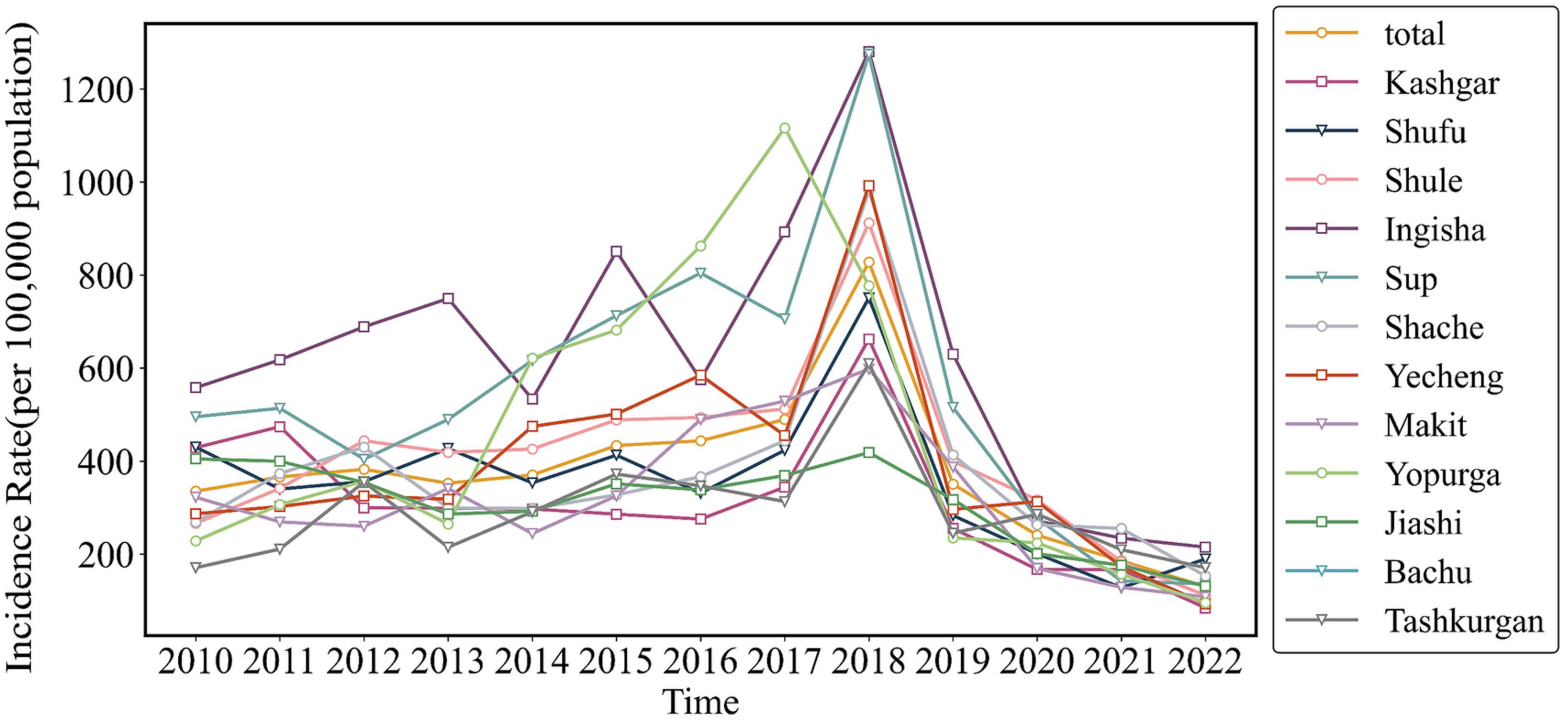
Trend of tuberculosis incidence in Kashgar, Xinjiang Uygur Autonomous Region, 2010–2022.

### Spatial correlation analysis of air pollution and incidence of pulmonary tuberculosis

**Figure 8:**
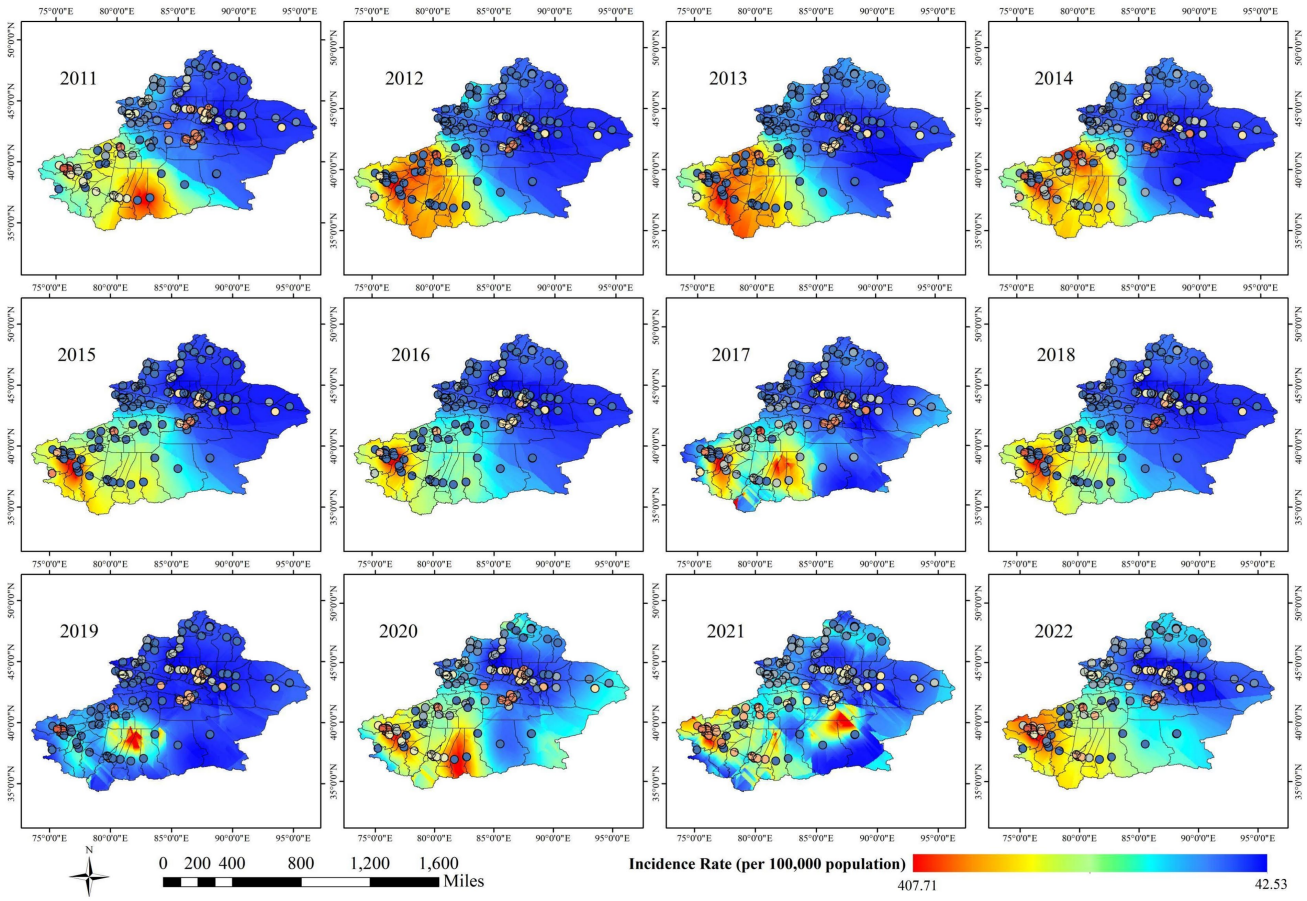
Spatial distribution characteristics of pulmonary tuberculosis incidence in Xinjiang Uygur Autonomous Region from 2011 to 2022.

Figure 8 shows the spatial distribution of pulmonary tuberculosis incidence in the Xinjiang Uygur Autonomous Region from 2011 to 2022. The southern Tarim Basin, surrounded by the Tianshan Mountains (including Kashgar, Hotan area, Aksu, and other areas of southern Xinjiang), has higher incidence rates, while the northern Zhungeer Basin (including kelamayi, Urumqi, Changjihuizu Autonomous Prefecture, and other areas of northern Xinjiang) shows relatively lower rates. This may be related to factors such as higher population density, relatively limited medical resources, and environmental and climatic conditions in southern Xinjiang, leading to significant regional differences in incidence rates between southern and northern Xinjiang.

Due to non-normal distribution and unequal variances in tuberculosis incidence (Levene statistic = 3.144, *p* = 0.03355), a Kruskal-Wallis test was performed, revealing significant regional differences (*p* < 0.0001). Dunn-Sidak post-hoc comparisons showed significantly higher tuberculosis incidence in Southern Xinjiang compared to Northern Xinjiang (Table 2).

**Table 2:** Dunn-Sidak multiple comparison results for tuberculosis incidence.

Group 1	Group 2	Estimate (lower bound, upper bound)	*p*-value
Northern	Southern	−431.83 (−483.59, −380.07)	0
Northern	Eastern	82.58 (−26.85, 192.01)	0.19973
Southern	Eastern	514.41 (406.14, 622.67)	0

**Figure 9:**
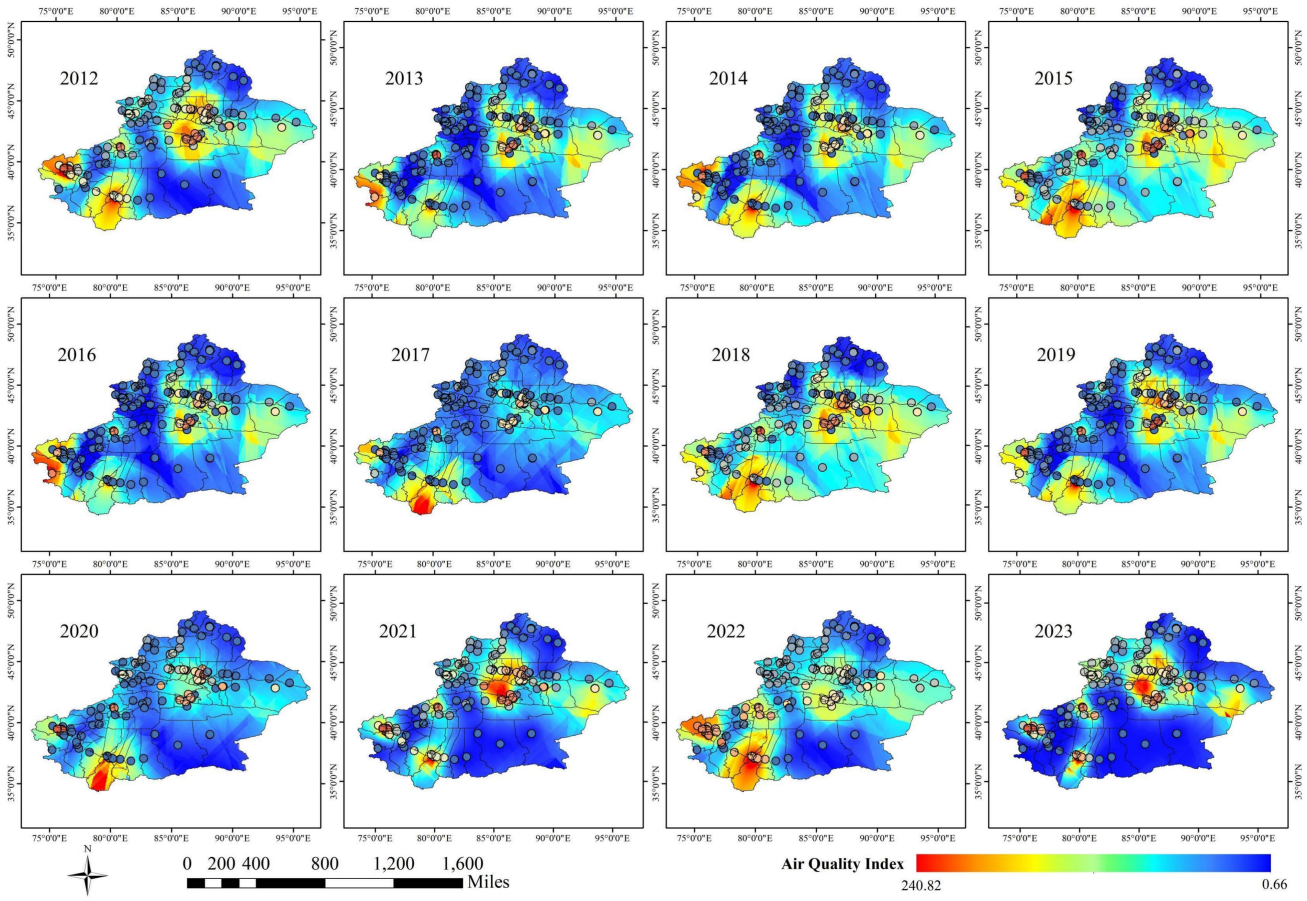
Spatial distribution characteristics of AQI in Xinjiang Uygur Autonomous Region from 2012 to 2023.

Meanwhile, Figure 9 illustrates the spatial distribution of AQI, which largely aligns with the spatial distribution of tuberculosis incidence. Southern Xinjiang, including Kashgar, Hotan area, and Aksu, experiences heavier pollution, while northern Xinjiang, such as Urumqi and Hami, sees relatively lighter pollution.

Notably, Urumqi, located in the central region of Xinjiang and surrounded by the Tianshan Mountains, shows higher tuberculosis incidence at specific sites according to Kriging interpolation, although the overall spatial analysis classifies the area as blue, indicating lower incidence. This contrasts with the AQI distribution, where Urumqi is marked red due to high pollution. This discrepancy is likely due to the uneven distribution of monitoring stations. Urumqi, a valley city near the Gurbantunggut Desert (semi-fixed), located in the northern foot of the middle section of the Tianshan Mountains and the southern margin of the Junggar Basin, is hindered by Mountain range that limit pollutant dispersion, resulting in consistently poor air quality. Despite its high pollution, Urumqi and Hami, unlike Kashgar and Hotan, are not directly impacted by desert dust. The overall pattern indicates that high tuberculosis incidence in southern Xinjiang, particularly in deeper areas of the Kunlun and Tianshan mountain regions, may be influenced by windblown desert particulate matter, as suggested by the hotspot areas in Figure 2. This supports the trend of southern Xinjiang being a high-risk area for tuberculosis, while northern Xinjiang remains relatively low-risk, as reflected in both Figures 8, 9.

This phenomenon may be influenced by various factors. It is important to note that these analyses are based solely on the available data and represent hypothetical interpretations. The actual situation may be affected by multiple factors, including but not limited to local environmental policies, industrial development, population density, increased industrial activities, or insufficient public health measures. The Kruskal-Wallis test revealed no statistically significant differences in AQI across regions (*χ^2^* = 4.722, *p* = 0.1933), suggesting that regional variations in AQI are not significant.

**Figure 10:**
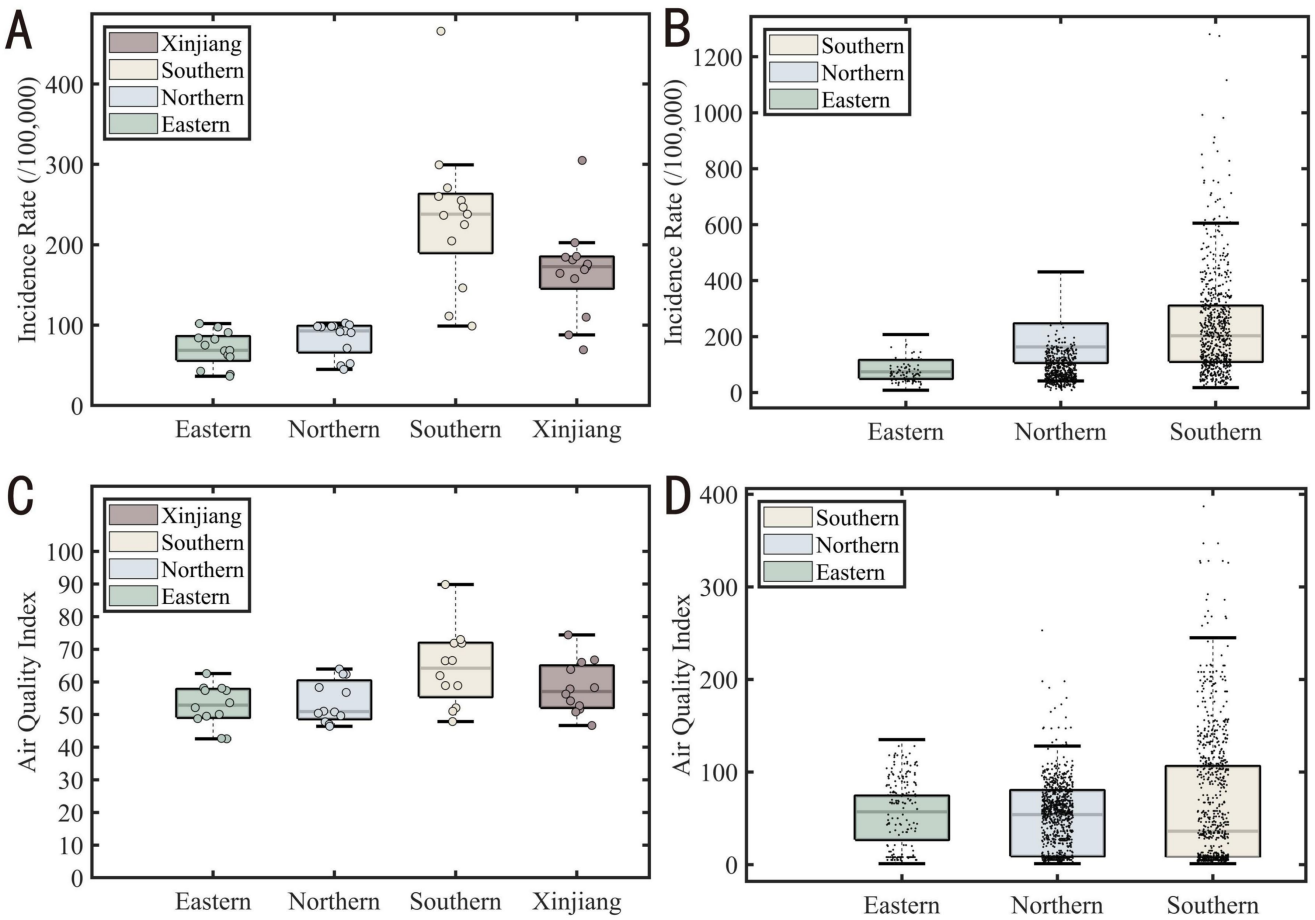
Boxplot comparison of tuberculosis incidence and air quality index (AQI) across Xinjiang regions. **(A)** Boxplot of regional tuberculosis incidence **(B)** Boxplot of tuberculosis incidence at county level **(C)** AQI box plot at regional level **(D)** AQI box plot at county level.

The Kruskal-Wallis test revealed significant differences in tuberculosis incidence, but AQI did not show a corresponding significant difference, despite following similar regional patterns. This may be due to the uneven distribution of monitoring stations, with more stations in rural and suburban areas compared to urban centers, leading to reduced statistical power in urban regions. Tuberculosis monitoring stations are concentrated in hospital districts, while AQI monitoring stations are mostly located in suburban areas, and the scarcity of urban stations may affect AQI’s statistical results. By including all available stations rather than random sampling, we enhanced the reliability of the spatial analysis. Southern Xinjiang, particularly regions like Kashgar, Hotan, and Aksu, showed higher tuberculosis incidence and AQI values, suggesting a potential correlation between air quality and health outcomes. These areas, surrounded by mountains and the Taklamakan Desert, may experience higher levels of particulate matter, contributing to both elevated AQI and tuberculosis rates. In contrast, Northern and Eastern Xinjiang exhibit better air quality and lower tuberculosis incidence, likely due to fewer environmental stressors such as desert dust. These spatial patterns are shown in Figure 10.

### Cold hot spot analysis

**Figure 11:**
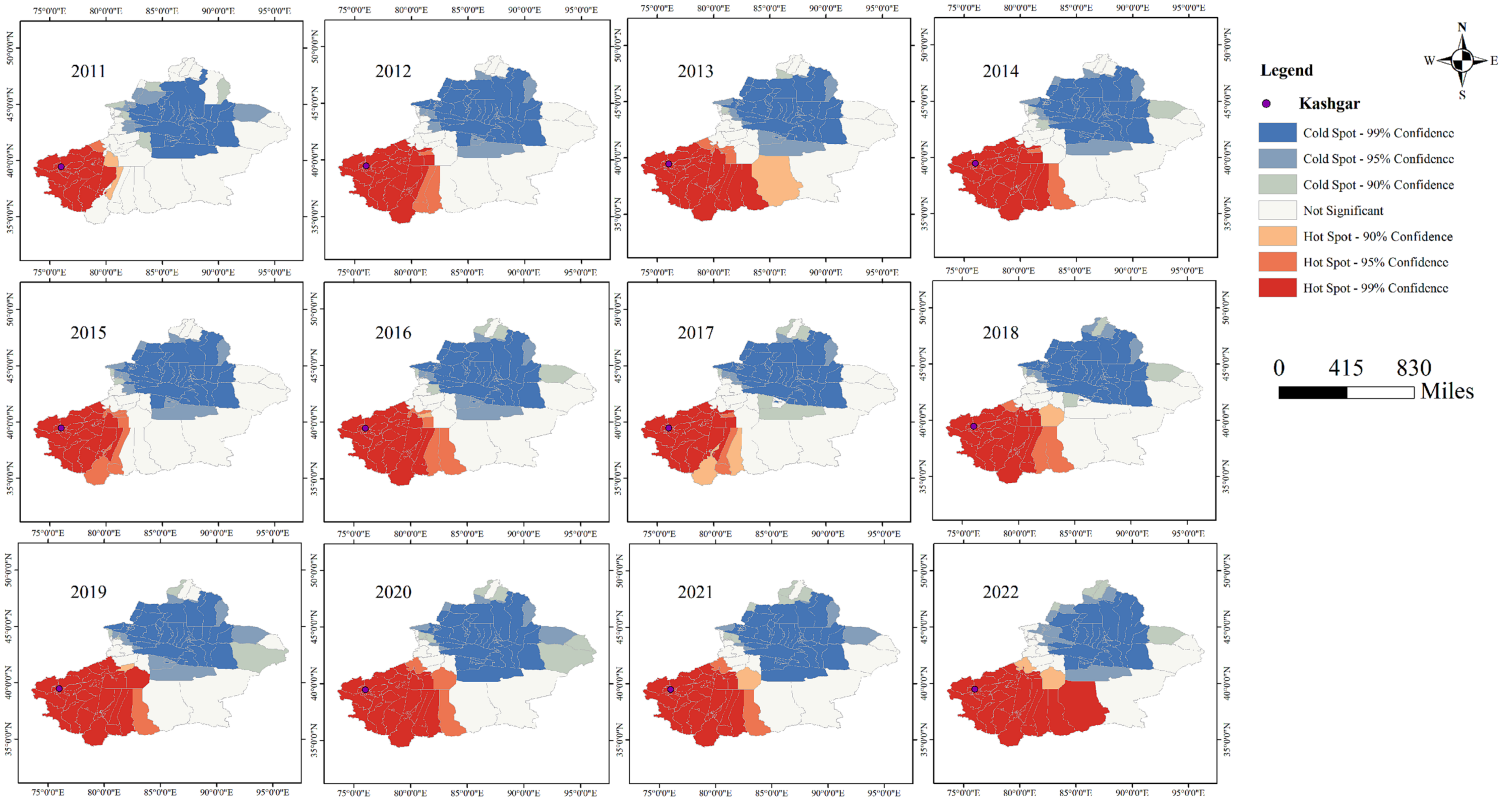
Cold hot spot analysis of pulmonary tuberculosis incidence in Xinjiang Uygur Autonomous Region from 2011 to 2022.

**Figure 12:**
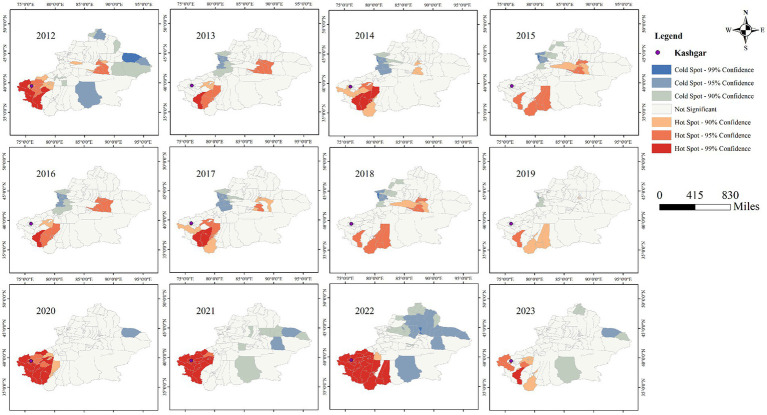
Cold hot spot analysis of AQI in Xinjiang Uygur Autonomous Region from 2012 to 2023.

Figures 11, 12 clearly show the differences between southern and northern Xinjiang through hot and cold spot analyses. The spatial hotspots for both AQI and tuberculosis incidence are located in southern Xinjiang, particularly in Kashgar, Hotan, and Aksu, which are surrounded by mountains on three sides and face the Tarim Basin and the Taklamakan Desert (migratory). The cold spots are mostly found in northern Xinjiang or other sparsely populated areas. Notably, in Figure 10, the AQI hotspots include the Urumqi area (located in the Tianshan mountain pass, in the central part of the Xinjiang map), whereas Figure 9 shows no corresponding tuberculosis hotspots in this region. This could be due to the significant spatial variability in tuberculosis distribution, where smaller Individual cases fail to influence the overall pattern of hot and cold spots.

### Cluster analysis of aerosol transmission trajectory

**Figure 13:**
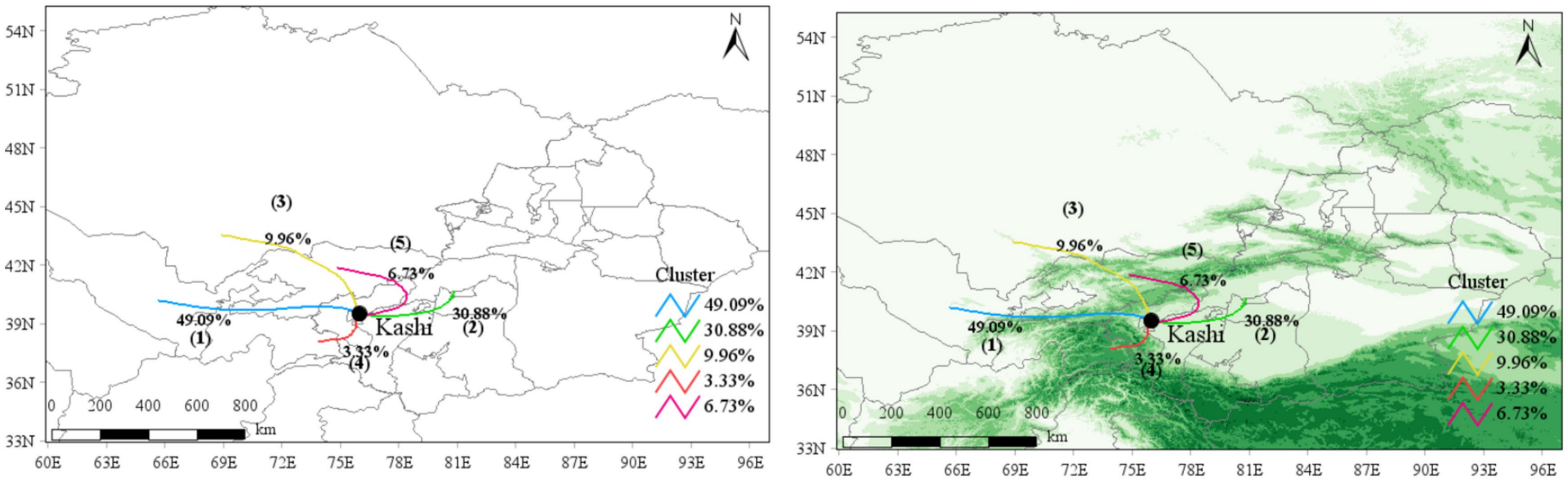
The intersection of the western part of the Tianshan mountains and the western part of the Kunlun mountains: a topographic map of the backward clustering trajectory in Kashgar [the second quarter (April–June) of 2015–2022].

Trajectory ① (blue line) in Figure 13 traverses from the northern junction of the West Tianshan Mountains to the southern junction of the West Kunlun Mountains, extending westward and eastward through Tajikistan to Kashgar. Trajectory ② (Green Line) extends from east to west, passing through the Aksu region, Bayinguolengmenggu Mongol Autonomous Prefecture, and Hotan area region to Kashgar in the west. Trajectory ③ (yellow line) extends from northwest to southeast, passing through Kyrgyzstan and the Kezilesukeerkezi Kyrgyz Autonomous Prefecture to Kashgar. Trajectory ④ (red line) extends from southwest to northeast, passing through Pakistan, including Islamabad, to Kashgar. Trajectory ⑤ (purple-red line) extends from northeast to southwest, passing through Kyrgyzstan and the Aksu region to Kashgar.

The Kashgar region experiences increased susceptibility to dust during the second quarter of each year. Trajectories originating from the Taklimakan Desert contribute 30.88 and 6.73% to Trajectories ② and ⑤, respectively, transporting a significant amount of dust aerosols to the Kashgar region. The proportion of PM_10_ pollution trajectories reaches 59.73 and 65.79%, with average trajectory concentrations of 500.72 and 471.47 ug/m^3^, respectively, higher than those in other directions, indicating substantial influence of dust on PM_10_ pollution levels in the second quarter. Trajectory 4 exhibits the shortest airflow transmission trajectory, primarily influenced by the southern Kunlun Mountains, leading to limited pollutant diffusion and accumulation of atmospheric pollutants, with higher average concentrations of PM_2.5_ and PM_10_. Trajectories ① (49.09%) and ③ (9.96%) cover longer transmission distances, originating mainly from Tajikistan and Pakistan, with lower pollution concentrations. Further details are reported in Table 3.

**Table 3:** Meteorological clustering trajectories of PM_2.5_ and PM_10_ in Kashgar from April to June in 2015–2022.

Trajectories (%)	PM_2.5_				PM_10_			
	Number	Mean (ug/m^3^)	Pollution number (%)	Mean pollution trajectory (ug/m^3^)	Number	Mean (ug/m^3^)	Pollution number (%)	Mean pollution trajectory (ug/m^3^)
1 (49.09)	1,362	54.48	25 (18.65)	171.20	1,361	181.59	455 (33.43)	402.25
2 (30.88)	819	142.05	407 (49.69)	251.22	817	500.72	488 (59.73)	784.55
3 (9.96)	282	55.66	54 (19.15)	173.20	282	195.67	91 (32.27)	452.57
4 (3.33)	91	84.05	37 (40.66)	154.61	91	250.97	50 (54.95)	389.67
5 (6.73)	191	128.78	98 (51.31)	217.46	190	471.47	125 (65.79)	672.01
All (100)	2,745	86.88	850 (100)	214.25	2,740	300.55	1,209 (100)	587.72

Notably, the main wind direction and the direction of topographic barriers are consistent with the previous hot and cold spot results, as well as the spatial correlation results. The meteorological cluster extending deeper into the desert has the largest proportion.

### Prediction of potential source area of tuberculosis

**Figure 14:**
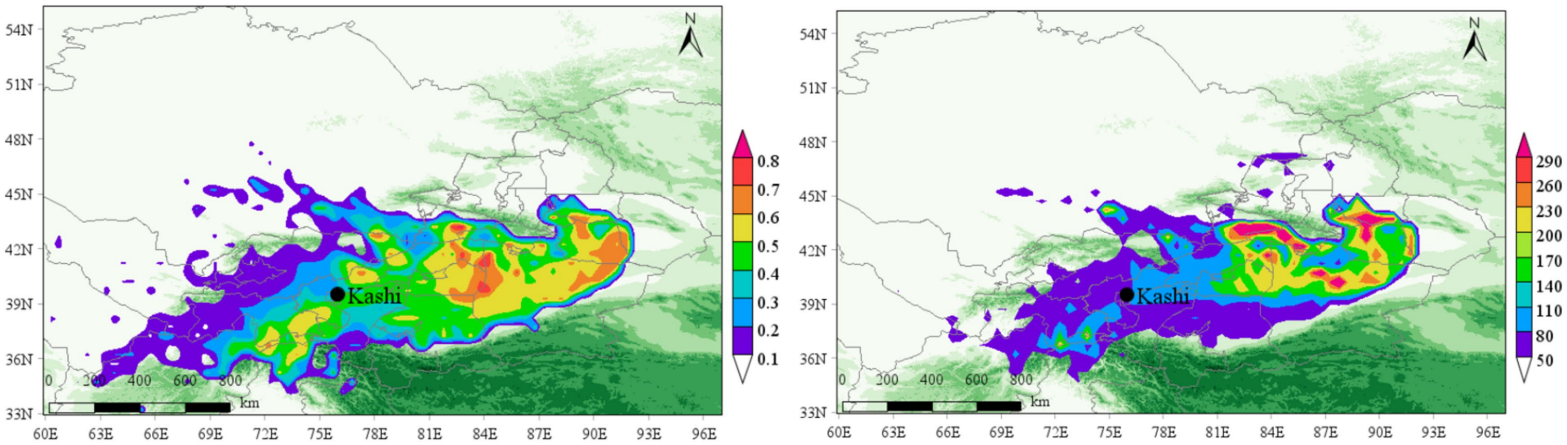
PSCF and CWT analysis of PM_2.5_ in Kashgar region in the second quarter of 2015–2022 (April–June).

**Figure 15:**
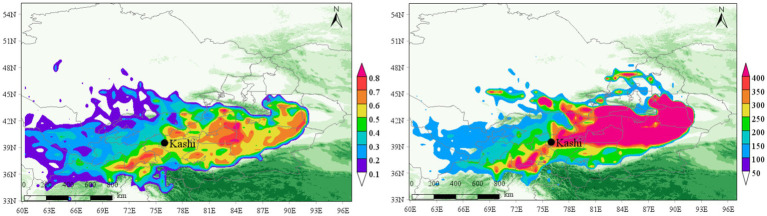
PSCF and CWT analysis of PM_10_ in Kashgar region in the second quarter of 2015–2022 (April–June).

The Weighted Potential Source Contribution Function (WPSCF) and Concentration Weighted Trajectory (WCWT) analysis provide insights into the influence of potential aerosol source areas on the Kashgar region. Figures 14, 15 present the PSCF and CWT analyses for PM_2.5_ and PM_10_ concentrations during the second quarter (April–June, which coincides with the peak incidence period for tuberculosis in this region.) from 2015 to 2022.

For PM_2.5_, the predominant pollution sources are located in the northeast, with severe pollution clusters concentrated in Aksu, Alar, Korla, and Urumqi, primarily originating from the Taklimakan Desert. The airflow patterns disperse moderate and mild pollution clusters toward the southwest and west. This is critical for epidemiology, as aerosols, including PM_2.5_, can facilitate the suspension of pathogens such as *Mycobacterium tuberculosis*, allowing them to remain airborne longer due to their attachment to fine particles. The combination of dust, air pollution, and the presence of resistant pathogens increases the risk of tuberculosis (TB) transmission, especially given Kashgar’s high population density and crowded living conditions.

The trajectory analysis for PM_2.5_ shows that WCWT values exceed 150 μg/m^3^ in regions such as Aksu, Alar, and Hotan, with peak values reaching 300 μg/m^3^ in some areas. This indicates a significant influence of aerosols in southwestern China on the region’s air quality and their potential role in facilitating the spread of TB.

In contrast, PM_10_ pollution is widely dispersed but severe in specific areas. Notably, the southern region of Kashgar, particularly areas affected by the Kunlun Mountains and located on the leeward slope, experiences significant pollution accumulation due to the blockage of air masses. WCWT values for PM_10_ exceed 400 μg/m^3^ in the northwest Taklimakan Desert and reach over 250 μg/m^3^ in the southern region of Kashgar, where the Kunlun Mountains contribute to the buildup of pollution. This geographic and meteorological pattern correlates with the high incidence of tuberculosis in Kashgar, demonstrating distinct regional characteristics.

These findings highlight the critical interaction between air pollution (specifically PM_2.5_ and PM_10_), the geographic environment, and tuberculosis incidence in Kashgar. The unique topographic and environmental factors exacerbate pollution levels, particularly in regions adjacent to major deserts and mountainous barriers, further intensifying the potential for airborne transmission of *Mycobacterium tuberculosis*.

## Discussion

The complex impacts of air pollution on human immunity involve inflammatory responses, oxidative stress modulation, and respiratory health (36–38). Fine particulate matter (PM_10_, PM_2.5_) has gained considerable attention for harboring pathogens like *Mycobacterium tuberculosis* that can facilitate their spread among populations (39). Studies have shown that exposure to pollutants, such as particulate matter (PM_2.5_), can alter immune system function, weakening the body’s defense mechanisms against tuberculosis and thus increasing transmission risk (40). In arid and semi-arid regions, environmental factors such as low humidity, high temperatures, and dust storms play a significant role in tuberculosis (TB) transmission. Recent studies have shown that in desert climates, such as those in Central Asia, the dry conditions and dust storms contribute to the prolonged survival of *Mycobacterium tuberculosis* in the air, increasing the risk of airborne transmission (41). Additionally, research comparing desert and temperate climates suggests that areas with better air quality and higher humidity tend to have lower TB transmission rates due to reduced airborne bacterial survival (42, 43). Although microscopic analyses have revealed potential links between tuberculosis and air quality (44), current research has not fully addressed how particulate matter propagates or interacts with geographic context, demographic health effects, and transmission mechanisms (45, 46). This study uses an epidemiological approach with geographical and meteorological data to analyze dust transmission, but our ability to trace *Mycobacterium tuberculosis* spread is limited by the lack of strain-based evidence, making any claims about tuberculosis transmission speculative and requiring further investigation.

When examining the relationship between air pollution and tuberculosis, several uncertainties must be taken into account. These include variations in air pollution measurement methods and the selection of pollutants such as PM_2.5_, nitrogen oxides, and ozone, which can influence the findings (47). The socioeconomic characteristics of the study population, especially in low-income areas with high levels of air pollution and tuberculosis incidence, complicate the determination of whether air pollution serves as an independent risk factor (48, 49). Although studies control for confounders like smoking, nutrition, population density, and healthcare access, these factors may still affect results (50). Tuberculosis (TB) is a chronic disease with a long incubation period, making it crucial to account for the time lag when analyzing the relationship between air pollution and TB. Future research should apply models such as Distributed Lag Nonlinear Models (DLNM) to conduct a more detailed analysis of time-lagged cumulative effects in the spatial distribution of diseases, especially by interpreting the latency period between exposure and disease onset. This approach will help clarify the relationship between air pollution and TB and reduce potential biases arising from lag effects (51, 52). Geographic location and climate conditions can also influence how this relationship manifests across regions (53). Additionally, demographic factors such as age, gender and socioeconomic status across regions may further influence tuberculosis incidence (54, 55). While Xinjiang’s population structure has stabilized under government policies, healthcare in some regions, especially in southern and northern Xinjiang, remains underdeveloped (56). Our study relies on crude incidence rates, which may affect the results (57).

The Kashgar region of Xinjiang is surrounded by mountains on three sides (58). The southern part of the northern Tianshan Mountains lies horizontally, and the Pamir Plateau stands in the west. The southern part is the Karakoram Mountains stretching from east to west, and the eastern part is the vast Taklimakan Desert (59). The dominant wind direction is easterly wind, which causes floating dust to stay in this area for a long time (60). The AQI shows a seasonal trend, with higher levels in the spring and winter and lower levels in the summer and autumn (61). In spring and summer, there are more blowing sand and dust storms, resulting in severe air pollution (62). At the same time, the incidence of tuberculosis exhibits two peaks throughout the year: one during the winter AQI peak and another during April and May, when sandstorms are most frequent (63). The terrain of the three mountains and two basins is prone to heavy pollution deposition. The southwesterly wind in the high altitude area prevents the diffusion of PM_2.5_ and PM_10_ to the southwest to a certain extent (64). It is blocked by the Tianshan Mountains, Kunlun Mountains and Pamirs. The spatial diffusion of pollutants and the potential source area are perpendicular to the mountains, which also verifies the deposition of particulate matter in Kashgar with a high incidence of tuberculosis. The strong correlation between pollutant distribution, tuberculosis incidence, and pollution source characteristics supports the impact of pollution on tuberculosis (65, 66). This study found significant differences in pulmonary tuberculosis incidence and atmospheric particulate matter concentration between southern and northern Xinjiang, providing evidence to support previous research (12). While the direct causal link between these pollutants and specific health outcomes like tuberculosis is not fully established by our study, it is reasonable to hypothesize that such an accumulation could potentially contribute to the observed health trends in the region (67, 68). Due to Kashgar’s unique environmental conditions, there is no better evidence of the impact of atmospheric particulate matter and dust transmission on tuberculosis incidence than in this area (69).

## Conclusion

Spatial and temporal analysis using Kriging interpolation and time series observation reveals a significant overlap between high tuberculosis (TB) incidence areas and regions with poor air quality in southern Xinjiang, divided by the Tianshan Mountains. Hot and cold spot analysis further confirms this pattern. Specifically, the case study of dust transmission in Kashgar highlights the role of atmospheric pollutants in influencing TB incidence in this unique environment. Inhalation of particulate matter impairs immune function, increasing disease susceptibility, while providing an environment conducive to *Mycobacterium tuberculosis* proliferation. The accumulation of airborne pollutants is strongly linked to higher TB incidence in the region. Trajectory analysis and meteorological clustering reveal pathways and source areas for pollutants, showing that atmospheric particulate matter significantly contributes to the increased risk of dust-borne TB in Kashgar. Although this phenomenon is evident, direct epidemiological evidence, such as bacterial strain analysis, is lacking to confirm TB transmission via these particulates. The hypothesis that particulate matter carries *Mycobacterium tuberculosis* remains speculative but plausible given the environmental conditions. To reduce health risks, residents should avoid outdoor activities, especially strenuous exercise, during high pollution periods to limit exposure to harmful particles. Policymakers should focus on measures like sandstorm prevention, afforestation, land reclamation, and wetland protection to address the environmental factors worsening air quality.

## Limitations

Our study has several limitations: (1) There is no strain-based epidemiological evidence or experimental proof indicating that these particulates carry *Mycobacterium tuberculosis* from polluted source areas to high-incidence regions. Therefore, this article merely describes local phenomena and presents possible speculations. (2) The unevenness in our data reflects the historical development of air quality stations in Xinjiang, with stations increasing over time and the earliest records from 2012. Prior to that, few national testing stations existed, limiting spatial analysis. Additionally, lag effects between TB and air quality must be considered. (3) Our research focuses solely on macro-level statistical analysis of time and space, neglecting micro-level experimental and methodological research. (4) We exclusively consider environmental factors such as medicine and meteorology, overlooking sociological factors like population dynamics and potential confounding variables. The incidence rate of crude tuberculosis is affected by many factors. (5) The annual levels of airborne pollutants and tuberculosis incidence are shown; however, there is no statistical analysis proving the quantitative impact of air pollution on the disease.

## Data Availability

The original contributions presented in the study are included in the article/Supplementary material, further inquiries can be directed to the corresponding author.

## Footnotes

^1^ http://www.cnemc.cn/

^2^ http://data.Cma
