# Supplementary material for: Interactions among tuberculosis, geographic environment and aerosols: evidence from the Kashgar region of China
Source: Front Public Health. 2025 Mar 19;13:1519330. doi: 10.3389/fpubh.2025.1519330 (PMC11961933; doi:10.3389/fpubh.2025.1519330)
Supplement: Supplementary file 1 [file Table_1.docx]

The correlation analysis results revealed the relationship between AQI and tuberculosis incidence across four regions (Eastern, Northern, Southern, Xinjiang). Spearman correlation was used for all regions. Specifically, the Spearman correlation for the Eastern region was 0.1238 (*P*=0.687), indicating a weak and non-significant relationship. For the Northern region, the Spearman correlation was -0.0989 (*P*=0.7507), suggesting a non-significant negative correlation. In the Southern region, the Spearman correlation was -0.1018 (*P*=0.7407), showing no significant negative correlation. For Xinjiang, the Spearman correlation was -0.1264 (*P*=0.6826), also weak and non-significant.(**Supplementary Table S1**).

**Supplementary Table S1:Correlation Analysis Results for AQI and Tuberculosis Incidence**

| Region | Correlation Methods | *Correlation Coefficient* | *P-value* |
| --- | --- | --- | --- |
| Eastern | Spearman | 0.1238 | 0.687 |
| Northern | Spearman | -0.098901 | 0.75073 |
| Southern | Spearman | -0.10179 | 0.74073 |
| Xinjiang | Spearman | -0.12637 | 0.68263 |
