# Supplementary material for: Interactions among tuberculosis, geographic environment and aerosols: evidence from the Kashgar region of China
Source: Front Public Health. 2025 Mar 19;13:1519330. doi: 10.3389/fpubh.2025.1519330 (PMC11961933; doi:10.3389/fpubh.2025.1519330)
Supplement: Supplementary file 2 [file Table_2.docx]

Regression analysis examined the effect of AQI on tuberculosis incidence. In the Eastern region, the regression equation showed an intercept of 41.921 and AQI coefficient of 0.51433 (*R²*=0.0766), indicating a weak effect of AQI on tuberculosis incidence. For Northern, the intercept was 89.267, AQI coefficient was -0.19779 (*R²*=0.0096), showing a minimal negative effect. In the Southern region, the regression result had an intercept of 285.82 and AQI coefficient of -1.1795 (*R²*=0.0204), also suggesting a small negative effect. In Xinjiang, the intercept was 247.98, AQI coefficient -1.6281 (*R²*=0.0785), indicating a weak negative impact(**Supplementary Table S2**).

**Supplementary Table S2: Regression Analysis of AQI on Tuberculosis Incidence by Region**

| Region | Intercept *(β0)* | AQI coefficient *(β1)* | *R-squared value* |
| --- | --- | --- | --- |
| Eastern | 41.92105947 | 0.51432511 | 0.07663291 |
| Northern | 89.26686322 | -0.1977931 | 0.009637502 |
| Southern | 285.816323 | -1.179462598 | 0.02037466 |
| Xinjiang | 247.9760365 | -1.628072888 | 0.078513631 |
